# Supplementary material for: Hydrothermal Crystallization of Bismuth Oxychlorides (BiOCl) Using Different Shape Control Reagents
Source: Materials (Basel). 2021 Apr 27;14(9):2261. doi: 10.3390/ma14092261 (PMC8123882; doi:10.3390/ma14092261)
Supplement: Supplementary file 1 [file materials-14-02261-s001.zip › materials-1150592-supplementary.pdf]

materials

Hydrothermal Crystallization of Bismuth Oxychlorides (BiOCl) Using Different Shape Control Reagents

Enikő Bárdos [1,2], Viktória A. Márta [1], Szilvia Fodor [1,2], Endre-Zsolt Kedves [3,4,5] and Klara Hernadi [1,6,*] and Zsolt Pap [1,2,4,*]

1 Department of Applied and Environmental Chemistry, University of Szeged, Rerrich Béla tér 1, HU-6720, Szeged, Hungary; bardosenci@gmail.com (E.B.), martaviktoria95@gmail.com (V.A.M.), fod_szilvia@hotmail.com (S.F.)
2 Institute of Environmental Science and Technology, University of Szeged, Tisza Lajos krt. 103, Szeged HU-6720, Hungary
3 Faculty of Physics, Babeș–Bolyai University, M. Kogălniceanu 1, RO–400084 Cluj–Napoca, Romania; kedves.endre91@gmail.com
4 Nanostructured Materials and Bio-Nano-Interfaces Center, Institute for Interdisciplinary Research on Bio-Nano-Sciences, Babeș-Bolyai University, Treboniu Laurian 42, RO–400271 Cluj-Napoca, Romania
5 Department of Biosystems Engineering, Faculty of Engineering, University of Szeged, Moszkvai krt. 9, H-6725 Szeged, Hungary
6 Institute of Physical Metallurgy, Metal Forming and Nanotechnology, University of Miskolc, 3515 Miskolc-Egyetemváros, Hungary
* Correspondence: hernadi@chem.u-szeged.hu (K.H.), pzsolt@chem.u-szeged.hu (Z.P.)

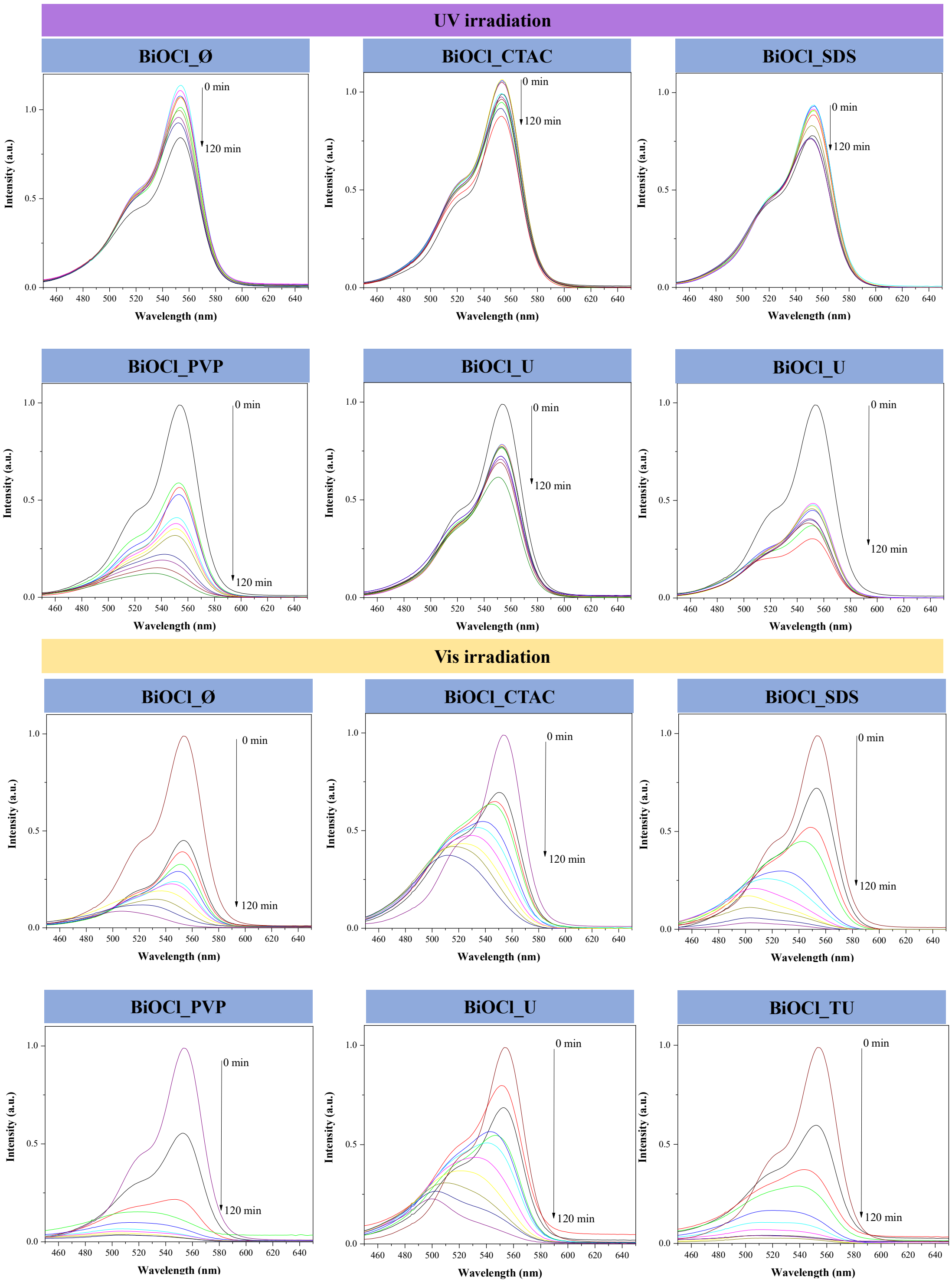

Figure S1. The RhB spectra of the Rhb degradation under UV and Vis irradiation
